# Delineating the Spectrum of Genetic Variants Associated with Bardet-Biedl Syndrome in Consanguineous Pakistani Pedigrees

**DOI:** 10.3390/genes14020404

**Published:** 2023-02-03

**Authors:** Ali Raza Rao, Aamir Nazir, Samina Imtiaz, Sohail Aziz Paracha, Yar Muhammad Waryah, Ikram Din Ujjan, Ijaz Anwar, Afia Iqbal, Federico A. Santoni, Inayat Shah, Khitab Gul, Hafiz Muhammad Azhar Baig, Ali Muhammad Waryah, Stylianos E. Antonarakis, Muhammad Ansar

**Affiliations:** 1 Molecular Biology and Genetics Department, Medical Research Center, Liaquat University of Medical and Health Sciences, Jamshoro 76090, Pakistan; 2 Institute of Basic Medical Sciences, Khyber Medical University, Peshawar 25100, Pakistan; 3 Department of Genetics, University of Karachi, Karachi 75270, Pakistan; 4 Scientific and Ophthalmic Research Laboratory, Sindh Institute of Ophthalmology and Visual Sciences, Hyderabad 71000, Pakistan; 5 Department of Pathology, Liaquat University of Medical and Health Sciences, Jamshoro 76090, Pakistan; 6 Department of Ophthalmology, University of Lausanne, Jules Gonin Eye Hospital, Fondation Asile Des Aveugles, 1004 Lausanne, Switzerland; 7 Department of Zoology, Lahore College for Women University, Lahore 54810, Pakistan; 8 Department of Genetic Medicine and Development, University of Geneva, 1211 Geneva, Switzerland; 9 Department of Endocrinology Diabetes and Metabolism, University Hospital of Lausanne, 1011 Lausanne, Switzerland; 10 Department of BioSciences, Faculty of Life Science, Mohammad Ali Jinnah University, Karachi 75400, Pakistan; 11 Department of Biotechnology, Institute of Biochemistry, Biotechnology and Bioinformatics, The Islamia University of Bahawalpur, Bahawalpur 63080, Pakistan; 12 iGE3 Institute of Genetics and Genomics of Geneva, 1211 Geneva, Switzerland; 13 Advanced Molecular Genetics and Genomics Disease Research and Treatment Centre, Dow University of Health Sciences, Karachi 74200, Pakistan

**Keywords:** retinitis pigmentosa, BBS, genetic variants, Pakistan

## Abstract

This study aimed to find the molecular basis of Bardet-Biedl syndrome (BBS) in Pakistani consanguineous families. A total of 12 affected families were enrolled. Clinical investigations were performed to access the BBS-associated phenotypes. Whole exome sequencing was conducted on one affected individual from each family. The computational functional analysis predicted the variants’ pathogenic effects and modeled the mutated proteins. Whole-exome sequencing revealed 9 pathogenic variants in six genes associated with BBS in 12 families. The *BBS6/MKS* was the most common BBS causative gene identified in five families (5/12, 41.6%), with one novel (c.1226G>A, p.Gly409Glu) and two reported variants. c.774G>A, Thr259LeuTer21 was the most frequent *BBS6/MMKS* allele in three families 3/5 (60%). Two variants, c.223C>T, p.Arg75Ter and a novel, c. 252delA, p.Lys85STer39 were detected in the *BBS9* gene. A novel 8bp deletion c.387_394delAAATAAAA, p. Asn130GlyfsTer3 was found in *BBS3* gene. Three known variants were detected in *the BBS1, BBS2*, and *BBS7* genes. Identification of novel likely pathogenic variants in three genes reaffirms the allelic and genetic heterogeneity of BBS in Pakistani patients. The clinical differences among patients carrying the same pathogenic variant may be due to other factors influencing the phenotype, including variants in other modifier genes.

## 1. Introduction

Bardet-Biedl syndrome (BBS) is a rare autosomal recessive genetic disorder, with at least 26 genes reported to cause BBS in different ethnicities [1]. BBS shows variable intra-familial and inter-familial phenotypes; the clinical presentations may include retinal degeneration and strabismus, postaxial polydactyly, obesity, hypogonadism, intellectual disability, hepatic fibrosis, diabetic mellitus and speech deficits [2,3].

The BBS-associated genes encode numerous ciliary-associated proteins [4]. Primary cilia are responsible for photoreceptor function in the retina and permit the transport of molecules in photoreceptors. Cilia dysfunction causes retinal degeneration, renal diseases, obesity, cerebral anomalies, and diabetes [5]. Interaction of multiple BBS-associated genes forms a BBSome-complex, essential in cilia formation. For example, the *BBS6, BBS10*, and *BBS12* proteins form a chaperonin complex, which acts with *BBS7*, *BBS2*, and *BBS9* to form the core of the BBSome protein complex [6].

The prevalence of BBS is variable among different world populations. The highest incidence, 1/3700, has been reported in the Faroe Islands [7], followed by 1/17,000 and 1/18,000 in Newfoundland and Kuwaiti populations [8,9]. BBS is rare in the European population and its prevalence varies from 1/125,000 to 1/160,000 in English and Swiss populations [10,11], whereas it is even lower at 1 in 18 million in the Asian population [12]. The prevalence of BBS is not well defined in the Pakistani population; only 20 affected families belonging to different ethnic groups have been reported. Further comparative studies are needed to explore the genetic pattern of BBS in the Pakistani population.

The Pakistani population is genetically heterogeneous and the fraction of consanguineous marriages is higher than in other countries. 60% of marriages in the country are consanguineous [13], resulting in increased autosomal recessive disorders. This study aimed to determine the molecular cause of BBS in consanguineous families and further study the phenotypic heterogeneity.

## 2. Materials and Methods

### 2.1. Enrollment of Participants

The Bioethics Committee approved this study of the University Hospitals of Geneva, Geneva, Switzerland (Protocol number: CER 11–036) and the Research Ethics committee of Liaquat University of Medical & Health Sciences, Jamshoro, Pakistan. Informed written consent was obtained from all participants. Clinical examination confirmed the BBS-associated phenotypes, and family history was recorded. Twelve consanguineous families with a minimum of two affected siblings with BBS belonged to different ethnic groups and regions of Pakistan. The LUBS-1 to LUBS6, LUBS-9, and LUBS-10 families were enrolled from different cities of Sindh province and belonged to mixed ethnic groups. The CB-3 and CB-44 families have enrolled from Khyber Patktoon Khuwa (KPK) region, and both originate from the same ethnic group. Both the RP-04 and VI-44 families were enrolled from Punjab province and belonged to the same ethnic group.

### 2.2. Blood Sampling and Clinical History

Pedigrees of all families were drawn, and family history was recorded. Detailed clinical information was taken, and BBS-related phenotypic characteristics, including postaxial polydactyly, brachydactyly, obesity, nystagmus, strabismus, intellectual disability, and obesity, were noted in every affected and normal individual (Table 1, Supplementary Figure S1). Then, 10 mL blood sample was collected. DNA was extracted by using a standard optimized protocol [14].

### 2.3. Whole Exome Sequencing Data Analysis

Whole exome sequencing (WES) was performed at the University Hospital Geneva, Geneva, Switzerland using SureSelect Human All Exon kit v5 (Agilent Technologies, Santa Clara, CA, USA) on an Illumina HiSeq4000 [15]. Exome data were analyzed through a customized pipeline, and we successfully used the following strategy to identify novel ID/DD genes in consanguineous families [15,16]. The pipeline includes the Burrows–Wheeler aligner tool (BWA), SAMtools, PICARD (http://broadinstitute.github.io/picard/ (15 June 2020)) and GATK [17]. The human assembly GRCh37/hg19 was used for reference alignment [18]. WES was performed in one affected member per family to an overall mean-depth base coverage of at least 100-fold, and >90% of the targeted region covered at least 20-fold. Mapping of sequenced reads and variant calling was performed as described previously [15,16,19]. First, variants in the genes reported to cause BBS and/or retinitis pigmentosa (RP) were extracted from the WES variant files to look for genetic diagnosis. Extracted variants were filtered with a minor allele frequency <1% in the GnomAD [20] and our local database. The remaining variants were prioritized according to (i) their predicted deleteriousness scores calculated by the SIFT [21], PolyPhen [22] and MutationTaster [23], (ii) GERP scores [24] to look at the conservation, (iii) the severity of the genetic alteration (e.g., truncation vs missense vs synonymous variant). Cases in which we found pathogenic or likely pathogenic variants in known BBS/RP genes were further investigated by genotyping all family members through Sanger sequencing for the segregation of variants with the disease phenotypes in corresponding families [25].

The human assembly GRCh37/hg19 was used for reference alignment [18]. WES was performed in individuals; IV-8 of LUBS01, IV-3 of LUBS02, IV-1 of LUBS03, IV-2 of LUBS09, IV-2 of LUBS05, IV-1 of LUBS06, IV-1 of RP-04, IV-4 of VI-65, IV-4 of CB03 and IV-2 of CB04. Additionally, both parents and all siblings of all families were genotyped for the found variants by sanger sequencing.

### 2.4. Sanger Sequencing Method

Sanger sequencing was performed at the Department of Molecular Biology and Genetics, Liaquat University of Medical and Health Sciences, Jamshoro Sindh, Pakistan. Primer pairs were designed to amplify variants using the Primer 3 web tool (Supplementary Table S1). The sequencing reaction was carried out for affected families and 60 normal controls using the previously described Big dye terminator Sanger sequencing kit [16]. The samples were electrophoresed using Genetic analyzer 3130, and the chromatograms were analyzed using chromas ver 3.

### 2.5. Computational Analysis for Protein Predictions

Bioinformatic analysis was performed for in silico predictions of pathogenic variants and their effects on the encoded protein. For non-synonymous substitutions, Polyphen2 [26], Sift, Mutation taster, and HOPE (Have your Protein Explained) protein prediction web tool were used and Provean, and Editseq were used for Frameshift variants [22,23,27]. The Phyre2 bioinformatics tool was used to model the protein [28].

## 3. Results

Twelve unrelated consanguineous families with more than two affected siblings with BBS were enrolled from Sindh, Punjab, and KPK provinces of Pakistan. The medical examination confirmed the clinical diagnosis of BBS with variable clinical presentations, showing interfamilial and intra-familial phenotypic differences. The exome sequencing revealed 9 likely causative variants; four novels and five reported in six different genes; these variants were segregated with the disease phenotype in all 12 families. Two variants were detected in the *BBS9* (NM_198428.3) gene, including one nonsense substitution (c.223C>T, p.Arg75Ter) and a second novel single base deletion, resulting in a frameshift followed by premature termination (c.252delA, p.Lys85SerTer39). An 8bp novel deletion, c.387_394delAAATAAAA, pAsn130GlyfsTer3 was found in *BBS3* (NM_001278293.3) gene and one novel substitution 1226G>A, p.Gly409Glu was identified in *BBS6/MKKS* (NM_170784.3) gene. The variants were homozygous in each family studied. No disease alleles were detected in 120 ethnically matched normal controls.

Five variants were previously reported, including a frameshift deletion (c.774delA, Thr259LeuTer21), a missense substitution (c.748G>A, Gly250Arg) in *BBS6/MKKS* (NM_170784.3), one nonsense substitution (c.1150G>T, p, Glu384Ter) in *BBS1*, one splice site variation (c.471G>A) in *BBS2* (NM_031885.5), and a 3bp inframe deletion (c.580_582delGCA, p.A194del) in *BBS7* (Supplementary Figure S2). The in silico functional studies supported the pathogenic role of all variants found segregating with the BBS phenotype in the study (Supplementary Table S2).

**Figure 1:**
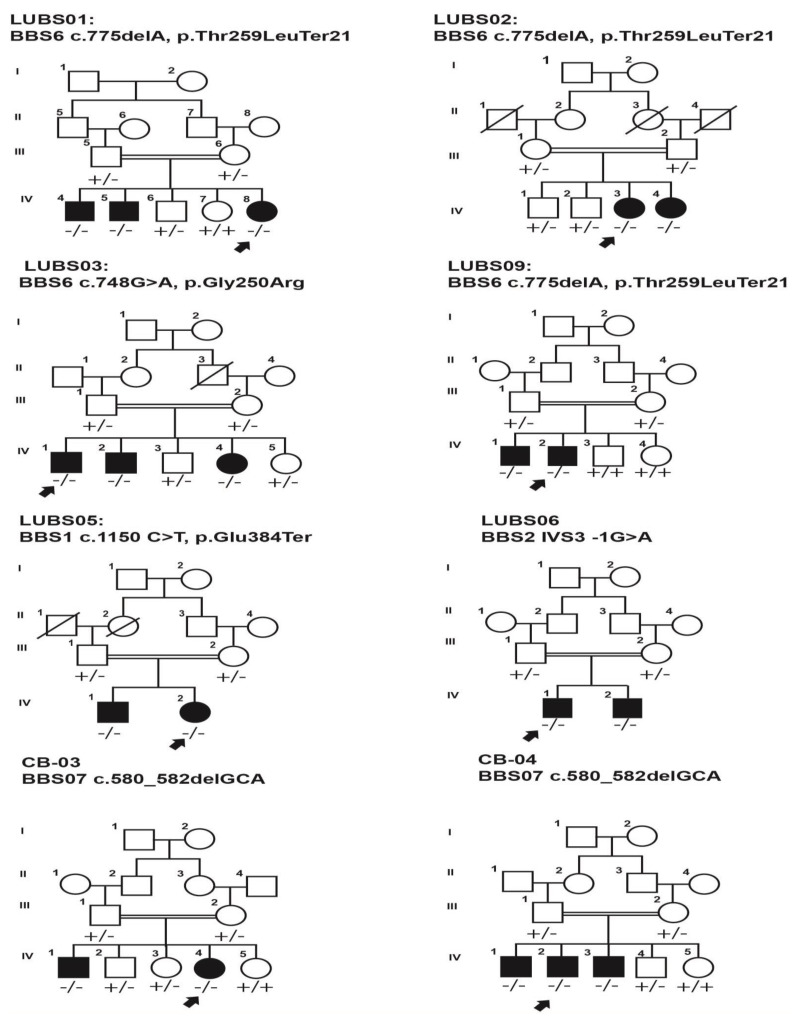
Pedigrees affected with Bardet-Biedl Syndrome carrying known variations in BBS genes.

The *BBS6*/MKKS was the most common causative *BBS* gene in the study (41.6%, 5/12 families) and the c.774delA:p. (Thr259LeuTer21) was the frequent *BBS6/MKKS* variant found in three families (60%, 3/5). All five families harboring *BBS6/MKKS* variants were consanguineous. The three families (LUBS-01, LUBS-02, and LUBS-09) carrying c.774delA:p.(Thr259LeuTer21) were unrelated and belonged to different ethnic groups (Figure 1). The clinical presentation of patients is described in Table 1; briefly, the age of the patients carrying c.774delA:p. (Thr259LeuTer21) ranged from 6 to 17 years, with a mean age of 12. There were three female and five male patients. The retinitis pigmentosa and obesity were consistent phenotypic features among all affected individuals, whereas polydactyly showed interfamilial and intra-familial variability. It is noteworthy that intellectual disability was observed in all three patients of LUBS09. In contrast, five patients of the other two families had normal intellectual development. Two patients of LUBS01 and LUBS09 had hypogonadism, each belonging to LUBS01 and LUBS09. The protein prediction showed that adenine deletion causes the frameshift and only results in a premature polypeptide of 259 amino acids. Another BBS6/MKKSreported missense variant c.748G>A, p. (Gly250Arg) segregated with BBS in family LUBS-03 (Figure 1). This family belonged to the Pakhtoon ethnic group of Mirpur Khas, Sindh, and consisted of two affected members, a boy and a girl, aged 12 and five years, respectively. Both patients had polydactyly at birth, whereas the retinitis pigmentosa and obesity manifested at three years. The in silico analysis revealed that this substitution (p.Gly250Arg) replaces glycine, which is neutral and small in size, whereas arginine is positively charged and big. This replacement of the amino acids might affect the binding function of the apical domain of the *BBS6/MKKS* protein.

**Figure 2:**
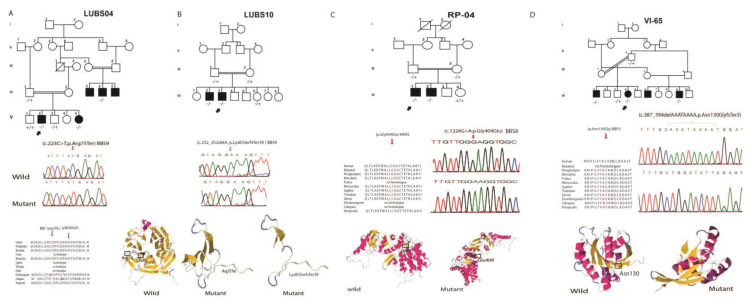
Bardet-Biedl Syndrome affected Pedigrees with novel variations showing chromatogram along with the wild type and mutant protein models.

Two patients of the RP-04 family were carrying a novel substitution, resulting in a missense variant c.1226G>A, p.Gly409Glu (Figure 2C). Both patients had night blindness as the primary symptom, and only one (IV:01) presented with polydactyly. In silico analysis showed that the wild and mutant amino acid differs in size, charge, and hydrophobicity. The mutant residue is bigger than the wild-type residue; the wild-type residue is more hydrophobic than the mutant residue. The wild-type glycine is the most flexible of all residues. This flexibility might be necessary for the protein’s function. Mutation of this glycine can abolish this function. Mutation of a 100% conserved residue is usually damaging to the protein. The Proven, Polyphen and mutation tester tools indicate the change as harmful and disease-causing. In addition, this variant is not found in the genomeAD database.

**Figure 3:**
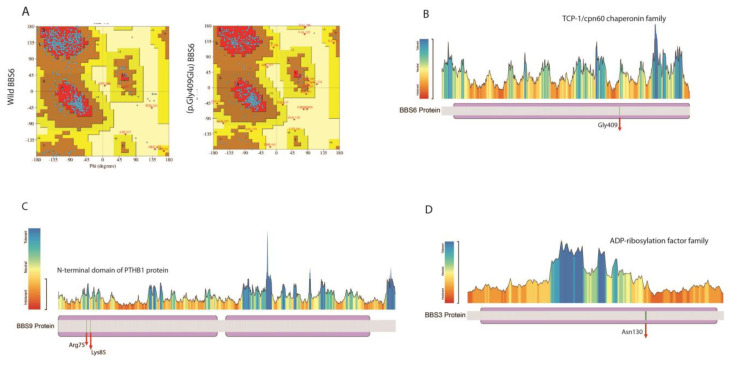
Protein modeling and bioinformatics analysis of identified variants. (**A**) Ramachandran plot for wild-type and mutated residues of the BBS6/MKKS gene. (**B**) MetaDome health map showing BBS6/MKKS residue. (**C**) MetaDome health map showing BBS9 residues. (**D**) MetaDome health map showing BBS3 residue.

The Ramachandran plot was used to predict the effect of amino acid substitution on protein structure. It compares the stereo-chemistry and geometry of wild and mutant types of protein structure by analyzing the angles of amino acids. The wild type and mutant proteins revealed a non-comparable range. The wild-type protein carried 82% and 17% residues in favored and allowed regions, while the mutant structure had 87% and 8% residues in favored and allowed regions (Figure 3A). The metaDome health map shows that the glycine at 409 position is located in the TCP-1/cpn60 chaperonin family domain and is found in the neutral region (Figure 3B).

A novel 8bp deletion c.387_394delAAATAAAA, resulting in truncation of the protein p.Asn130GlyfsTer3 in the *BBS3* gene (NM_001278293.3), was segregated with BBS in the VI-65 family (Figure 2D). The clinical examination of three patients showed that retinitis pigmentosa was the consistent phenotype in all the patients, whereas only one had polydactyly. The deletion of 8 nucleotides removes conserved amino acids and results in a non-homologous sequence. (Figure 2D). The deletions may disturb the small GTP-binding protein domain and GTP hydrolysis activity of *the BBS3* gene. The metaDome analysis revealed that Asn 130 amino acid is located in an intolerant region of ADP-ribosylation (Figure 3D).

A previously reported *BBS1* (NM_024649.5) variant, c.1150G>T: p.(Glu384Ter), was found segregating with BBS in two affected individuals of family LUBS-05 (Figure 1). The family belonged to the Pathan ethnic group and was enrolled from Dadu, Sindh, Pakistan. Both affected individuals were diagnosed with retinitis pigmentosa, obesity, polydactyly, and hypogonadism. The affected boy (IV-1) also manifested intellectual disability. The c.1150G>T substitution introduces premature stop codon at the 384th residue of BBS1 protein, affecting the apical domain and impairing binding properties.

Two truncating mutations were detected in the *BBS9* (NM_198428.3) gene, segregating with BBS in two unrelated families (Figure 2A,B). LUBS04 consisted of four affected individuals who belonged to the Sindhi ethnic group (Figure 2A). All the affected individuals had retinitis pigmentosa as the primary phenotype, polydactyly, and obesity. None of the affected family LUBS04 had intellectual disability and hypogonadism. The exome sequencing revealed nonsense codon in homozygosity, c.223C>T, p.Arg75Ter; the truncated protein is only 75 amino acids long and lacks the functionally important conserved Pfam domain. This allele was previously reported in Danish and Saudi cohorts [29,30]; however, no details of this variant’s clinical and in silico functional data are available in the literature. Our bioinformatics analysis supported this change as disease-causing. The metaDome analysis revealed that the truncated Arg75 amino acid is located in the neutral region of the N-terminal domain of the PTHB1 protein (Figure 3C).

The second novel homozygous variant c.252delA, p.Lys85SerTer39 in the *BBS9* resulted in a frameshift and truncation of the protein at the 39th amino acid, segregating with BBS in family LUBS10 (Figure 2B). The affected individuals had consistent typical symptoms, including RP, polydactyly, and obesity. In addition, both affected were intellectually disabled. This variant affects the conserved Pfam domain of the BBS9 protein. The bioinformatics analysis indicated this variation as disease-causing. The metaDome analysis revealed that truncated Lysine at the 85th position is located in the slightly intolerant region of the N-terminal domain of the PTHB1 protein (Figure 3C).

One known splicing variant, IVS3 -1G>A, was found in the *BBS2* gene, which segregated with the BBS phenotypes in two affected individuals of family LUBS06. Both affected had RP, obesity, and intellectual disabilities, whereas polydactyly was absent in both patients (Table 1). The family belonged to the Punjabi ethnic group and was enrolled from Sindh province. The splice variant is predicted to cause the failure of removal of intron-3, resulting in abnormal protein.

A 3bp deletion (c.580_582delGCA:p(Ala194del)) in *BBS7* (NM_176824.3) gene segregated with the BBS phenotype in two unrelated families, CB03 and CB44. Both families were enrolled from the KPK province of Pakistan and belonged to the same ethnic group, Pashtun. The clinical findings showed that all five affected of the two families had RP, polydactyly, obesity, and nystagmus. In contrast, intellectual disability was found only in two affected individuals of family CB-44. Bioinformatics analysis showed that the deletion of the conserved Ala194 deteriorated the normal protein structure and function.

## 4. Discussion

In this study, we expand the repertoire of BBS phenotypes caused by the reported and novel variants in different BBS genes. We report 31 one affected individuals from 12 BBS families ascertained from different regions of Pakistan, who possess different pathogenic and likely pathogenic homozygous variants in BBS genes. The study affirmed the *BBS6/MKKS* alleles as the most common BBS-causing variants in Pakistani patients. (41.6% 5/12 families). The c.774delA was the frequent variant in the mutated 60% (3/5) BBS6/MKKSfamilies. The allele frequency of the frequent *BBS6/MKKS* allele, c.774delA in Pakistani patients was 24% (14/58) (Table 2). Our study showed a 41.6% (10/24 alleles) contribution of *BBS6/MKKS* alleles in the included families (Table 2). The global contribution is insignificant; only one family carrying the *BBS6/MKKS* mutation was detected in 55 families comprising European-derived American, Tunisian, Arabic, and Pakistani patients [31]. To date, 60 pathogenic variations have been reported in the *BBS6/MKKS* gene, most of which are missense and nonsense mutations [32]. Notably, the frequent mutation c.774delA was first detected in a Pakistani family. In contrast, the second missense variant found in the study, p.Gly250Arg was initially detected in a Spanish family [33]. This study identifies a novel *BBS6/MKKS* variant, p.Gly409Glu, indicating the allelic heterogeneity. A haplotype of intragenic variants across the c.774delA was assessed in families LUBS01, LUBS02 and LUBS09. A typical region of 3’033’925 bp was shared between family LUBS01 and LUBS02; LUBS09 shared a 2’636’377 bp region with LUBS01 and LUBS02, indicating a founder effect (Supplementary Table S3). Overall assessment of *BBS6/MKKS-*associated disease in Pakistani patients ranks it as a frequently mutated gene, with 27% (16/58) prevalence in the Pakistani patients studied. (Table 2).

*BBS9* variants are the second most frequent cause of BBS in Pakistan patients, with 20.6% (12/58) allelic contribution (Table 2). Previously, a single *BBS9* deletion, c.299delC, was found in three Pakistani families of the same ethnic group [33,39]. This study expands *the BBS9* mutation spectrum and adds two truncating variants c.223C>T, p.Arg75Ter and a novel c.252delA:p.Lys85SerTer39, associated with BBS in two unrelated pedigrees. The *BBS9* gene plays a central role in binding BBSome constituting proteins (BBS1 to BBS10), and the loss of function mutations may affect the integrity of the BBSome complex [6]. The affected of both families carrying novel variations presented three consistent phenotypic features, including RP, polydactyly, and obesity. In addition, LUBS10 patients had intellectual disability and hypogonadism (Table 1). The previously reported Pakistani families carrying a loss of function variant showed the same significant clinical symptoms, except for intellectual disability and hypogonadism. However, the two tested patients were adults aged 20 and 18 [38]. This phenotypic variation among the families reported in this study and previously reported pedigree may be due to different ethnic lineages.

Previously, three different mutations in the *BBS3* gene have been identified in three Pakistani families affected with BBS; one of the homozygous variants is a deletion of 54 Kb [31,40,41]. Notably, four other large deletions in *BBS3* were found in BBS families from Saudi Arabia, France and USA [37,38]. In this study, a novel 8 bp deletion (c.387_394delAAATAAAA) was inherited recessively in three patients of BBS. Initial clinical assessment of the proband indicated a nonsyndromic RP; however, the examination of all other affected showed unilateral polydactyly in one patient. Previously, nonsyndromic RP has been reported in Saudi Arabian patients carrying homozygous missense variants (p.Ala89Val) in *the BBS3* gene. In contrast, patients with a deletion, c.732+1952_899-3806-del4139, showed polydactyl, obesity, and dysmorphism [42]. The variable phenotype may be due to different types and locations of *BBS3* variants.

*BBS1* mutations are uncommon in Pakistani patients; previously, only one family with a splice site mutation was reported. We identified another BBS family carrying bi-allelic substitution p.Glu384Ter, resulting in the termination of the protein. Previously, c.1150G>T was detected as a compound heterozygous with a missense allele, Met390Arg, in French patients [42]. This study described the first report of homozygousp.Glu384Ter from Pakistan. The *BBS1* mutations are more frequent in Caucasian patients; the founder variant p.(Met390Arg) has been homozygous and compound heterozygous in more than 15 families [34,43]. In addition, *BBS1* variants cause mild ocular and renal abnormalities [6,34]. Our patients, homozygous for the *BBS1* variant, showed early onset of RP at 3 and 6 years and renal anomalies. These clinical differences may be due to the nature of the variant; the Caucasian patients harbored missense variants, whereas our patients carried bi-allelic truncation mutations. In addition, epigenetic or environmental factors may also aggravate the phenotype.

Identification of BBS mutation in the *BBS2* gene elaborates clinical and genetic heterogeneity in BBS patients of Pakistan. Previously no disease allele of *BBS2* has been reported in Pakistani patients. We detected a splice site variant, IVS3 -1G>A, in two patients of the LUBS06 family, which was initially reported in an isolated case affected with nonsyndromic retinitis pigments [44]. The affected individuals of family LUBS06 presented with typical characteristics and features of BBS, including polydactyly and obesity (Table 1). Our findings show that the IVS3 -1G>A segregates with BBS phenotype in a consanguineous pedigree, an additional variant phenotype.

*BBS7* mutation p.(Ala194del) segregated BBS into two families of the Pathan ethnic group. The clinical assessment showed interfamilial differences among patients despite the same variant and ethnicity. Two patients of the CB44 family had an intellectual disability, whereas no such phenotype was present in family CB03. This deletion was first reported in a Pakistani family of the same ethnicity, and the patients had RP, obesity, and intellectual disability [38]. Our study indicates that p.Ala194del is the recurrent BBS mutation of Pathan ethnicity and KPK province. The haplotype analysis showed a shared region of 5,063,463 bp long between the two families and indicated a common origin of the variant.

## 5. Conclusions

BBS is a rare disorder, and this is the largest cohort of consanguine BBS families, characterized by the molecular basis of the disease. The *BBS6/MKKS* and *BBS9* are the frequent genes associated with BBS in the Pakistani population, and identifying novel variants reaffirms the allelic heterogeneity. In addition, the differences in clinical manifestation and severity of the disease in patients carrying the same mutated allele indicate the contribution of other factors, including additional genomic variations. This study provides carrier screening and genetic counseling opportunities for affected families and helps in the prognosis and management of patients with BBS.

## Figures and Tables

**Table 1:** Clinical findings of the families affected with Bardet biedl syndrome.

Family Identity	Gene	Variants	Sex	Age (Years)	Retinitis Pigmentosa	Polydactyly	Intellectual Disability	Hypogonadism	Renal Failure	Obesity	Nystagmus	Deafness	Bone Deformity
LUBS-01	BBS6/MKKS	c.775delA, p.Thr259LeuTer21											
IV:4			M	12	Yes	Yes	No	Yes	No	Yes	No	No	No
IV:5			M	08	Yes	Yes	No	NA	No	Yes	No	No	No
IV:8			F	6	Yes	Yes	No	NA	No	Yes	No	No	No
LUBS-02	BBS6/MKKS	c.775-delA, p.Thr259LeuTer21											
IV:03			F	15	Yes	No	No	NA	No	Yes	No	No	No
IV:04			F	11	Yes	No	No	NA	No	Yes	Yes	No	No
LUBS-03	MKKS	c.748G>A, p.gly250Arg											
IV:01			M	12	Yes	Yes	No	NA	NA	Yes	Yes	No	No
IV:04			F	5	Yes	Yes	No	NA	NA	Yes	No	No	No
LUBS-04	BBS9	c.223C>T, p.Arg75Ter											
IV:03			M	21	Yes	Yes	No	No	No	Yes	No	No	No
IV:04			M	17	Yes	Yes	No	No	No	Yes	No	No	No
V:02			M	14	Yes	Yes	No	No	No	Yes	Yes	No	No
V:05			F	07	Yes	Yes	No	NA	No	Yes	Yes	No	No
LUBS-05	BBS1	c.1150 C>T,Glu384Ter											
IV:01			M	06	Yes	Yes	Yes	Yes	Yes	Yes	No	No	No
IV:02			F	03	Yes	Yes	No	Yes	Yes	Yes	No	No	No
LUBS-06	BBS2	c.471 +1G>A											No
IV:01			M	30	Yes	Yes	NA	Yes	No	Yes	Yes	No	No
IV:02			M	18	Yes	Yes	NA	Yes	No	Yes	Yes	No	No
LUBS-09	MKKS	c.775delA, p.Thr259LeuTer21											
IV:01			M	10	Yes	Yes	Yes	No	No	Yes	Yes	No	No
IV:02			F	14	Yes	Yes	Yes	Yes	No	Yes	Yes	No	No
IV:03			F	17	Yes	Yes	Yes	NA	NA	Yes	Yes	No	No
LUBS-10	BBS9	c.252delA, p.Lys85SerTer39											
IV:02			M	16	Yes	Yes	Yes	Yes	No	Yes	Yes	No	No
IV:03			M	18	Yes	Yes	yes	Yes	No	Yes	yes	No	No
CB-03	BBS7	c.580_582delGCA, p.Ala194del											
IV:01			M	30	Yes	Yes	No	NA	NA	Yes	Yes	No	No
IV:04			F	34	Yes	Yes	No	NA	NA	Yes	Yes	No	No
CB-44	BBS7	c.580_582delGCA,p.Ala194del											
IV:01			M	11	Yes	Yes	No	NA	NA	Yes	Yes	No	No
IV:02			M	22	Yes	Yes	Yes	NA	NA	Yes	Yes	No	No
IV:03			M	21	Yes	Yes	Yes	NA	NA	Yes	Yes	No	No
VI-65	ARL6	c.387_394delAAATAAAA											
IV:01			M	19	Yes	No	No	No	No	No	No	No	No
IV:04			F	10	Yes	No	No	No	No	No	No	No	No
IV:06			M	12	Yes	Yes	No	No	No	Yes	No	No	No
RP-04	MKKS	1226G>A,p.Gly409Glu											
IV:01			M	20	Yes	Yes	No	No	No	No	No	No	No
IV:02			M	25	Yes	No	No	No	No	No	No	No	No

**Table 2:** BBS associated variants detected in Pakistani patients.

Gene	Variants	No. of Families	No. of Alleles	Frequency (gnomAD Database)	References
*BBS1*	c.1150G>T,p.Glu Ter384	1	2	0	[34]
	c.47 +1G>T	1	2	0.00000399	[35] [35]
	c.442 G>A,p.Asp148Asn	1	2	0.00029	
*BBS2*	c.471 +1G>A	1	2	0	[36]
*BBS3/* *ARL6*	c.534A>G.p.Gln178Gln	1	2	0.00000796	[37]
	c.387_394delAAATAAAA	1	2	0	In this study
*BBS5*	c.734_744del,p.Glu245Gly Ter18	2	4	0	[37]
*BBS6/MKKS*	c.775delA, p.Thr259LeuTer21	4	8	0.0000438	[38]
	c.1226G>A,pGly409Glu	1	2	0	In this study
	c.287 C>T,p.Ala96Val	1	2	0	[39]
	c.748 G>A,p.gly250Arg	1	2	0.0000159	
	c.822 C>G,p.Ser40 *	1	2	0	[38]
*BBS7*	c.580_582delGCA	3	6	0	[38]
	c.1592_1592delTCCAG	1	2	0	
*BBS8*	c.1347G>C,p.Gln449His	1	2	0	[38]
*BBS9*	c.223C>T, p.Arg Ter75	1	2	0.0000199	In this study
	c.252delA, p.Lys85S Ter39	1	2	0	
	c.299delC (p.Ser100Leu Ter24)	3	06	0	[40]
	c.1789 C>T,p.Gln Ter597	1	2	0	[37]
*BBS10*	c.271_272insT	1	2	0.000579	[38]
*BBS12*	c.2014G>A,p.Ala672Thr	1	2	0.001102	[37]
*Total*		29	58		

## Data Availability

Date may be provided on the request.

## Supplementary Materials

The following supporting information can be downloaded at: https://www.mdpi.com/article/10.3390/genes14020404/s1, Figure S1: Phenotypic features of families affected with BBS; Figure S2: Chromatograms of common known variants; Table S1: Sequencing primers used for selected exons of BBS genes; Table S2: Predictions of bioinformatics tools for variants segregating in families affected with Bardet Biedal Syndrome (BBS); Table S3: Haplotype of interagenic variants across the BBS6/MKKS and *BBS7* common variants.
