# Supplementary material for: Delineating the Spectrum of Genetic Variants Associated with Bardet-Biedl Syndrome in Consanguineous Pakistani Pedigrees
Source: Genes (Basel). 2023 Feb 3;14(2):404. doi: 10.3390/genes14020404 (PMC9956862; doi:10.3390/genes14020404)
Supplement: Supplementary file 1 [file genes-14-00404-s001.zip › Supplementary Table S1.pdf]

Supplementary Table S1: Sequencing primers used for selected exons of BBS genes.

GENE	PRIMER	SEQUENCE
**BBS1**	**EXON 12**	TTCCCCAGGCCTGTCTCTAT
		AGGCCTAGCTCCAGAAGGAC
**BBS2**	**EXON 3**	TCAAAATCTGCTCAGTTTACTCAAA
		CACAAAACACGAATGAATGCT
**BBS3**	**EXON 7**	TGCCTAACAGTGTGACAGTATGTG
		TGTAAAAACCTACAATGCCACAA
**BBS6**	**EXON 3**	TCCACTGTATTACCTGGGATACTC
		GGCACAGGACAAGATCTACG
**BBS6**	**EXON 5**	TGTTCTTTTGTAATTAAAATCACACTC
		CAGTGTTAAAGGCTACAAGTTTACC
**BBS7**	**EXON 6**	ATGCAAGTTGTATTCGTAACCTAA
		AAAAGTTCAATAACTCGTGCTGT
**BBS9**	**EXON 4**	GAATTGTTTTGTTTACTCACAGTGG
		TGGGAAAATGTTAACCAAGACA
