# Supplementary material for: Delineating the Spectrum of Genetic Variants Associated with Bardet-Biedl Syndrome in Consanguineous Pakistani Pedigrees
Source: Genes (Basel). 2023 Feb 3;14(2):404. doi: 10.3390/genes14020404 (PMC9956862; doi:10.3390/genes14020404)
Supplement: Supplementary file 1 [file genes-14-00404-s001.zip › Supplementary Table S2.pdf]

Supplementary Table S2

Predictions of bioinformatics tools for variants segregating in families affected with Bardet-Biedl syndrome (BBS)

Family Identity	Genes	Variants	Provean	Sift	Polyphen2,	Mutation taster
**LUBS-01**<br>**LUBS-02**<br>**LUBS-09**	BBS6/ MKKS	c.775delA, p.Thr259LeuTer21	Deleterious	Not applicable	Disease causing	Not applicable
**LUBS-03**	BBS6/ MKKS	c.748G>A, p.gly250Arg	Deleterious	Damaging	Disease causing	Probably Damaging
**LUBS-04**	BBS9	c.223C>T, p.Arg75Ter	Deleterious	Not applicable	Disease causing	Not applicable
**LUBS-05**	BBS1	c.1150 C>T, p.Glu384Ter	Stop gain	Deleterious	Not applicable	Disease causing
**LUBS-06**	BBS2	c.471 +1G>A	Splicing error	Splicing error	Splicing error	Splicing error
**LUBS-10**	BBS9	c.252delA, p.Lys85SerTer39	Deleterious	Not applicable	Disease causing	Not applicable
**CB-03**<br>**CB-44**	BBS7	c.580delGCA, p.Ala194del	Deleterious	Damaging	Disease causing	Probably Damaging
**VI-65**	ARL6	c.387_394delAAATAAAA	Deleterious	Not applicable	Disease causing	Not applicable
**RP-04**	MKKS	1226G>A, p.Gly409Glu	Deleterious	Damaging	Disease causing	Probably Damaging
