# Supplementary material for: Delineating the Spectrum of Genetic Variants Associated with Bardet-Biedl Syndrome in Consanguineous Pakistani Pedigrees
Source: Genes (Basel). 2023 Feb 3;14(2):404. doi: 10.3390/genes14020404 (PMC9956862; doi:10.3390/genes14020404)
Supplement: Supplementary file 1 [file genes-14-00404-s001.zip › Supplementary Table S3.pdf]

Supplementary Table S3: Haplotype of intragenic variants across the *BBS6/MKKS* and *BBS7* common variants

MKKS(NM_170784.3): c.774delA, p.Thr259LeuTer21

Family ID	LUBS01	LUBS02	LUBS09
	rs575534	rs575534	
	rs652633	rs652633	
	rs524625	rs524625	
	rs683145	rs683145	
	**MKKS(NM_170784.3):c.774del**	**MKKS(NM_170784.3):c.774 del**	**MKKS(NM_170784.3):c.774 del**
	rs1051419	rs1051419	rs1051419
	rs1131695	rs1131695	rs1131695
	rs1431433	rs1431433	rs1431433
	rs243887	rs243887	

Families LUBS01 and LUBS02 share a common region of 3'033'925 bp

Family LUBS09 shares a region of 2'636'377 bp with families LUBS01 and LUBS02

BBS7(NM_176824.3):c.580_582del

Family ID	CB03	CB04
	rs3733526	rs3733526
	rs343192	rs343192
	rs34766411	rs34766411
	rs1397645	rs1397645
	rs3733559	rs3733559
	rs2276959	rs2276959

rs71599153	rs71599153
rs4370153	rs4370153
rs769242	rs769242
**BBS7(NM_176824.3):c.580_582del**	**BBS7(NM_176824.3):c.580_582del**
rs1396082	rs1396082
rs4833837	rs4833837
rs13134412	rs13134412
rs1472949	rs1472949
rs199504785	rs199504785
rs750377319	rs750377319

Families CB03 and CB04 share a haplotype of 5063463 bp long
