# Supplementary figures and images for: Delineating the Spectrum of Genetic Variants Associated with Bardet-Biedl Syndrome in Consanguineous Pakistani Pedigrees
Source: Genes (Basel). 2023 Feb 3;14(2):404. doi: 10.3390/genes14020404 (PMC9956862; doi:10.3390/genes14020404)

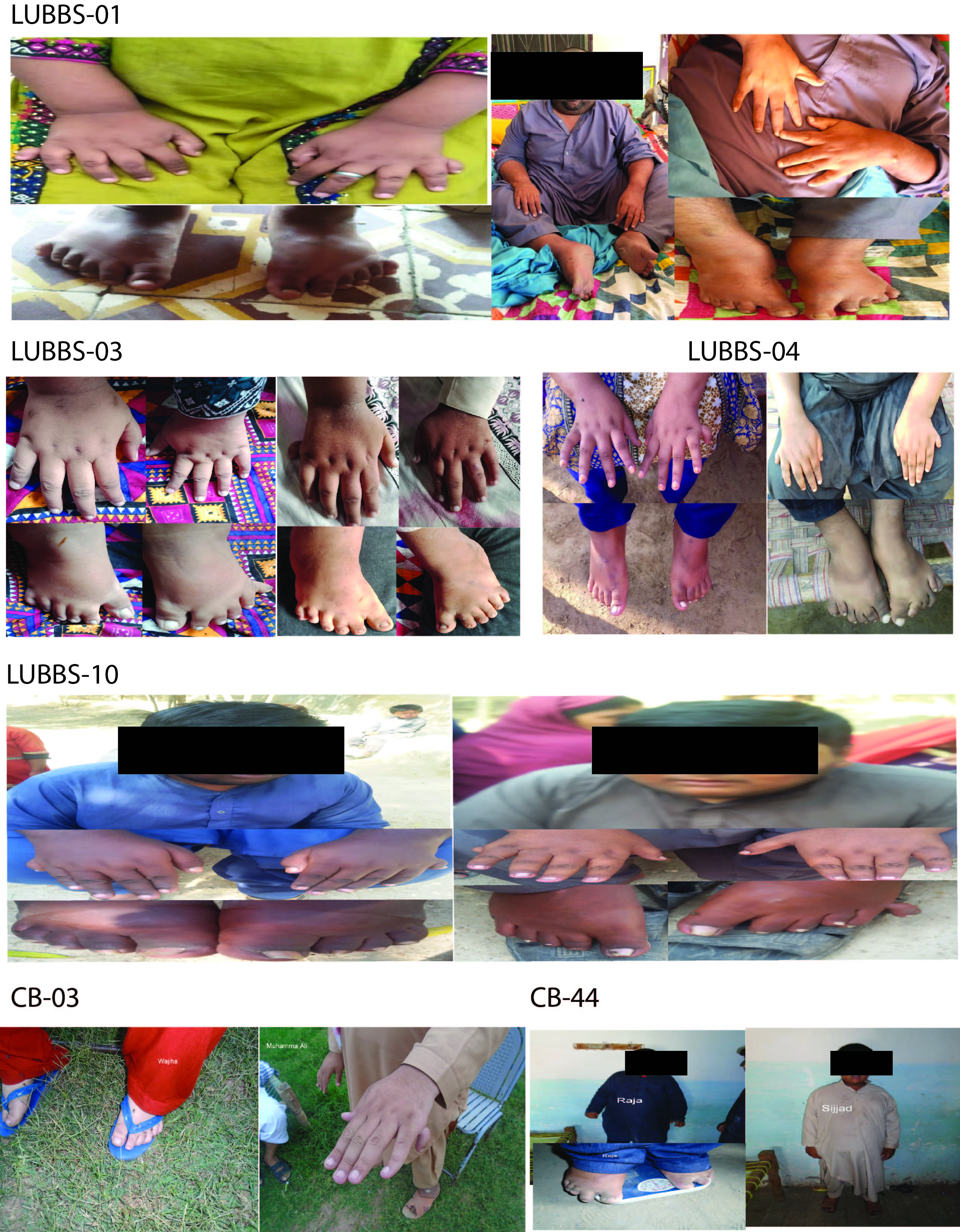

Supplement: Supplementary file 1 [file genes-14-00404-s001.zip › Supplementary Figure S1.jpg]

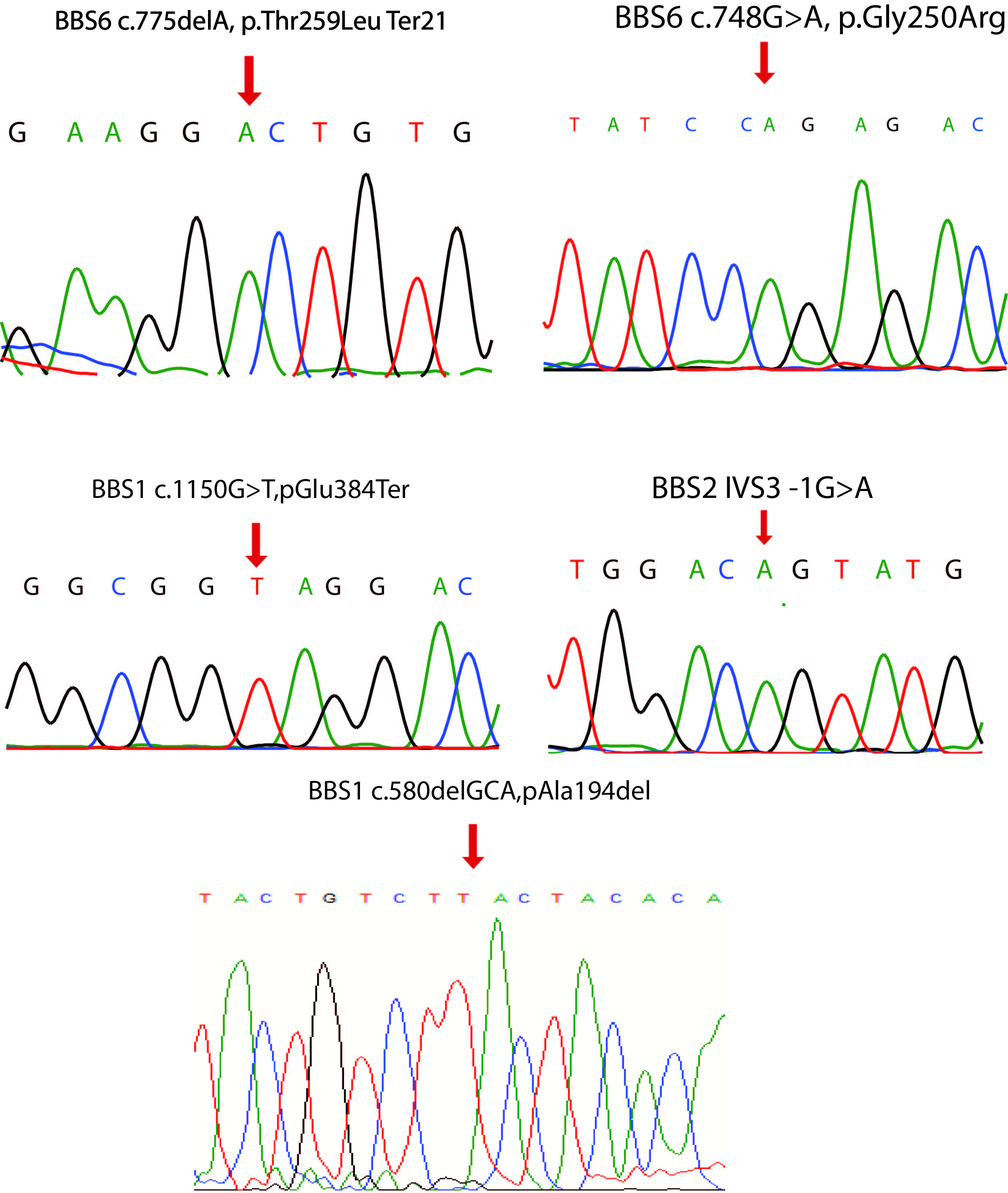

Supplement: Supplementary file 1 [file genes-14-00404-s001.zip › Supplementary Figure S2.jpg]
